# A Facile Synthesis and Characterization of Highly Crystalline Submicro-Sized BiFeO_3_

**DOI:** 10.3390/ma13133035

**Published:** 2020-07-07

**Authors:** Dovydas Karoblis, Diana Griesiute, Kestutis Mazeika, Dalis Baltrunas, Dmitry V. Karpinsky, Anna Lukowiak, Pawel Gluchowski, Rimantas Raudonis, Arturas Katelnikovas, Aleksej Zarkov, Aivaras Kareiva

**Affiliations:** 1 Institute of Chemistry, Vilnius University, Naugarduko 24, LT-03225 Vilnius, Lithuania; karoblisd@gmail.com (D.K.); diana.griesiute@chgf.vu.lt (D.G.); rimas.raudonis@chf.vu.lt (R.R.); arturas.katelnikovas@chf.vu.lt (A.K.); aleksej.zarkov@chf.vu.lt (A.Z.); 2 Center for Physical Sciences and Technology, LT-02300 Vilnius, Lithuania; kestas@ar.fi.lt (K.M.); dalis@ar.fi.lt (D.B.); 3 Scientific-Practical Materials Research Centre of NAS of Belarus, 220072 Minsk, Belarus; dmitry.karpinsky@gmail.com; 4 Institute of Low Temperature and Structure Research, Polish Academy of Sciences, Okolna 2, PL 50422 Wroclaw, Poland; a.lukowiak@int.pan.wroc.pl (A.L.); p.gluchowski@nanoceramics.pl (P.G.); 5 Nanoceramics Inc., Okolna 2, PL-50422 Wroclaw, Poland

**Keywords:** bismuth ferrite, BiFeO_3_, solution processing, magnetic properties

## Abstract

In this study, a highly crystalline bismuth ferrite (BFO) powder was synthesized using a novel, very simple, and cost-effective synthetic approach. It was demonstrated that the optimal annealing temperature for the preparation of highly-pure BFO is 650 °C. At lower or higher temperatures, the formation of neighboring crystal phases was observed. The thermal behavior of BFO precursor gel was investigated by thermogravimetric and differential scanning calorimetry (TG-DSC) measurements. X-ray diffraction (XRD) analysis and Mössbauer spectroscopy were employed for the investigation of structural properties. Scanning electron microscopy (SEM) was used to evaluate morphological features of the synthesized materials. The obtained powders were also characterized by magnetization measurements, which showed antiferromagnetic behavior of BFO powders.

## 1. Introduction

In the recent decades, bismuth ferrite (BFO, BiFeO_3_) has received great attention from the scientific community due to its outstanding physical properties. It is a material of interest due to its unique properties, one of which is undoubtedly its multiferroicity. BFO is the only single-phase perovskite compound that exhibits multiferroic behavior at room temperature, since it is simultaneously G-type antiferromagnetic and strongly ferroelectric [1]. Its Curie temperature is 1103 K and its Neel temperature is 643 K [2]. BFO is a rhombohedrally distorted ABO_3_ perovskite-type compound, with R3c space group (#161) and hexagonal lattice parameters a = 5.58 Å and c = 13.87 Å [3]. In the BFO structure 6s lone pair electrons of Bi^3+^ ions are responsible for the ferroelectricity, while the partially filled d orbital of Fe^3+^ results in magnetic ordering [4]. Because of its multiferroic properties BFO is a very attractive material for use in modern technological devices.

Another very broad and important area is photo-induced applications, where lead-free BFO is a more promising material in comparison to other ferroelectric oxides because of its relatively small band-gap, which allow it to utilize quite a large part of the sunlight spectrum [5]. Thus, it is widely used in photovoltaics [6]. Moreover, BFO was shown to be an efficient visible light photocatalyst for the degradation of organic pollutants, such as antibiotics and dyes [7,8,9]. It was successfully utilized both in its pure form and in the form of composites with other materials [10,11].

During the last decade, different synthetic approaches were employed for the preparation of BFO powders. For example, BFO has been synthesized by solid-state reaction [12], hydrothermal [13], co-precipitation [14], microwave-assisted solution combustion [15] and other methods [16,17,18]. Utilizing different synthetic procedures, BFO was synthesized in the form of bulk ceramics [19], thin films [20] and different nanostructures, such as nanofibers [21], nanocubes and nanorods [22], nanoplates [23], etc.

Despite the fact that a variety of synthetic procedures have been utilized, preparation of high-purity BFO products is still not an easy task because of the formation of commonly obtained Bi- or Fe-rich neighboring phases, such as Bi_2_O_3_, Bi_2_Fe_4_O_9_ or Bi_25_FeO_39_ [24,25]. Additional difficulties arise from the fact that BFO is characterized by poor thermal stability and Bi is known to be a highly volatile element. These peculiarities place additional limitations on synthesis. At the same time, it is well known that the presence of secondary crystal phases can influence the physical properties and performance of BFO-based materials and devices. Thus, there is still a need for a simple and cost-effective procedure for the preparation of phase pure BFO powders.

The main aim of the present study was to develop such a simple and reliable synthetic approach for the preparation of high-purity and highly crystalline BFO powders. For this purpose, 2-methoxy-ethanol was used in a dual role of complexing agent and solvent. Our proposed synthetic approach does not require adjustment of the pH value of the reaction mixture or addition of complexing agents [26,27,28]. Moreover, 2-methoxyethanol was previously shown as a well-suitable solvent for wet chemical synthesis of thin films due to its high dissolution capability and compatibility with metal nitrates [29]. In this work, the phase composition and purity of the products were estimated by X-ray diffraction analysis and Mössbauer spectroscopy. Morphological features were analyzed employing scanning electron microscopy. Magnetization of the BFO powders was analyzed as well.

## 2. Materials and Methods

### 2.1. Synthesis

For the synthesis of BFO powders bismuth(III) nitrate pentahydrate (Bi(NO_3_)_3_·5H_2_O, 98%, Roth, Karlsruhe, Germany) and iron(III) nitrate nonahydrate (Fe(NO_3_)_3_∙9H_2_O, ≥ 98%, Alfa Aesar, Haverhill, MA, USA) were used as starting materials. All chemicals and solvents were used without additional purification. Appropriate stoichiometric amounts of metal salts were dissolved in 2-methoxyethanol (C_3_H_8_O_2_, ≥ 99%, Roth, Karlsruhe, Germany) resulting in a 0.5 M solution of total metal ions. The obtained solution was mixed on magnetic stirrer at 60 °C for 1 h. Next, organic solvent was evaporated under constant stirring at 130 °C and residual substance was dried in the oven at 100 °C overnight. After this, dried brownish precursor powders were ground in an agate mortar and annealed at 550, 600, 650 and 700 °C for 5 h in air with a heating rate of 1 °C/min.

### 2.2. Characterization

The thermal decomposition of precursor gel was investigated by thermogravimetric and differential scanning calorimetry (TG-DSC) analysis using a STA 6000 Simultaneous Thermal Analyzer (Perkin Elmer, Waltham, MA, USA). Dried sample (5-10 mg) was heated from 30 to 900 °C at 10 °C/min heating rate in a dry flowing air (20 mL/min). Powder X-ray diffraction (XRD) analysis was performed using Ni-filtered Cu Kα radiation on a MiniFlex II diffractometer (Rigaku, The Woodlands, TX, USA) working in Bragg-Brentano (θ/2θ) focusing geometry. The data were collected within 2θ range from 20 to 60° with a step width of 0.02° and scanning speed of 1°/min. Lattice parameters were refined by the Rietveld method using the FullProf suite. Morphological features of the synthesized products were analyzed with a SU-70 field-emission scanning electron microscope (FE-SEM, Hitachi, Tokyo, Japan). Particle size distribution was estimated from SEM micrographs using ImageJ software (Jolla, CA, USA). Dependence of magnetization of samples on the strength of magnetic field were recorded using a magnetometer consisting of a SR510 lock-in amplifier (Stanford Research Systems, Gainesville, GA, USA), a FH-54 Gauss/Teslameter (Magnet Physics, Cologne, Germany) and a laboratory magnet supplied by a SM 330-AR-22 power source (Delta Elektronika, Eindhoven, The Netherlands). For magnetic measurements powdered sample (120 mg) was placed into a plastic straw of 5 mm in diameter with a sample height of approximately 5 mm. Mössbauer spectra were measured at room temperature in transmission geometry using a ^57^Co(Rh) source and a Mössbauer spectrometer (Wissenschaftliche Elektronik GmbH, Starnberg, Germany). Diffuse reflectance spectra were recorded at room temperature on an FLS980 fluorescence spectrometer (Edinburgh Instruments, Kirkton Campus, UK) equipped with an integration sphere coated with Teflon. Teflon was also used as a white standard. The excitation and emission bandwidths were 4.00 and 0.15 nm, respectively. Integration time was 0.2 s and step size 0.5 nm.

## 3. Results and Discussion

**Figure 1:**
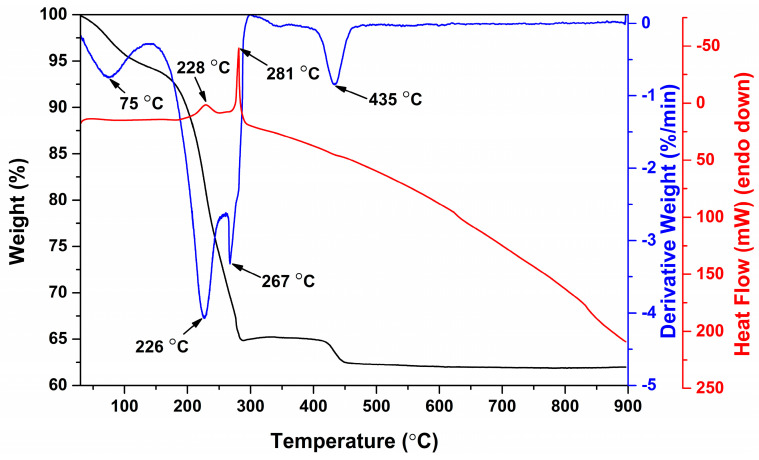
TG-DTG-DSC curves of BFO precursor gel.

Thermal analysis of the BFO precursor powder was performed in order to investigate its thermal decomposition behavior and determine the minimal annealing temperature at which all organic components of precursor decompose. TG-DTG-DSC curves of the dried BFO precursor are depicted in Figure 1. It is seen that degradation of the gel occurs in four main steps. The first stage is observed at low temperatures up to 130 °C. This weight loss could be ascribed to the removal of adsorbed water and volatile organic species. The most significant weight loss was observed in the temperature range from 150 to 300 °C, during this decomposition step 29% of the initial sample weight was lost. In this range two decomposition steps with the highest degradation rates at 226 and 267 °C can be clearly observed from DTG curve.

Each of these degradation steps is accompanied by exothermic peaks, which are centered at 228 and 281 °C, respectively. These signals could be attributed to the decomposition of organic part of the gel and combustion reaction between organic molecules and nitrates. The last decomposition step corresponding to the loss of around 3% of the weight occurred at 435 °C. This stage could be ascribed to the decomposition of residual organic components. At the same temperature range similar weight loss of approximately 2% was previously observed for sol-gel derived BFO precursor gel [26]. Residual mass was determined to be constant at temperatures above 450 °C and was equal to 62% of the gel mass.

**Figure 2:**
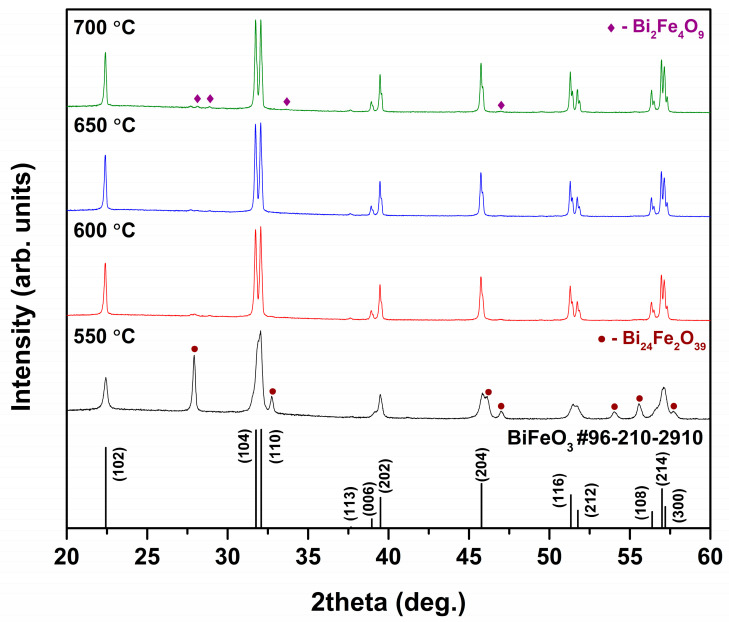
XRD patterns of BFO powders annealed at different temperatures.

Based on the results of thermal analysis it was determined that minimal annealing temperature required for the preparation of pure BFO powders is 450 °C. However, XRD analysis revealed that thermal treatment at such low temperature does not result in the formation of single-phase BFO. Figure 2 shows XRD patterns of BFO precursor gel annealed at different temperatures.

It is evident that even annealing at 550 °C was not sufficient for the formation of pure BFO phase. The observed reflections corresponding to BFO are very broad; moreover, high intensity additional peaks, ascribed to the secondary crystal phase, can be clearly seen at around 28°, 33°, 46°, 47°, 54°, 56°and 58°. This impurity was identified as Bi_24_Fe_2_O_39_ (PDF #00-042-0201). Annealing at higher temperatures prevented formation of this by-product and resulted in significantly purer BFO powders. It was determined that 650 °C is the optimal temperature for the preparation of high-purity BFO powders. However, even after a heat treatment at 650 °C negligible amount of neighboring Bi_2_Fe_4_O_9_ (PDF #00-072-1832) phase was observed. On the other hand, thermal treatment at 600 and 700 °C led to BFO product with still very insignificant amount of impurities. The position of diffraction peaks matched very well the position of those of standard XRD data (COD file #96-210-2910) for BFO with R3c space group. Lattice parameters of BFO sample prepared at 650 °C were calculated as a = b = 5.572 Å and c = 13.855 Å, which is in a good agreement with standard data. Moreover, the observed BFO peaks were very sharp indicating high degree of crystallinity of the powders and large grain size, since it is known that the width of diffraction peaks is inversely proportional to the grain size. Closer look at the XRD patterns revealed that the width of the diffraction peaks corresponding to BFO is independent of temperature in a studied range from 600 to 700 °C. Annealing at temperatures higher than 700 °C resulted in appearance of noticeable amount of Bi_2_Fe_4_O_9_ phase. The phase purity of the obtained BFO product clearly demonstrates the advantages of our suggested synthetic approach, since the fabrication of single-phase BFO remains a challenging task due to the formation of non-perovskite secondary phases. Many authors reported previously on the formation of significant amounts of neighboring phases, such as Bi_2_Fe_4_O_9_ or Bi_25_FeO_39_ [30,31,32,33]. Taking into account the results of XRD analysis, BFO sample annealed at 650 °C was chosen for further investigation.

**Figure 3:**
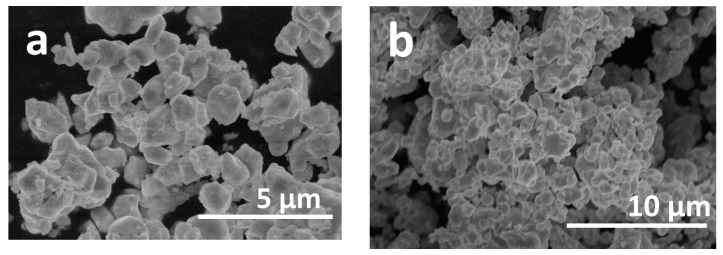
SEM micrographs of BFO powders annealed at 650 °C under different magnification (**a**) and (**b**).

SEM images of BFO powders annealed at 650 °C are given in Figure 3. The SEM image with higher magnification (Figure 3a) indicates that BFO powders consist of quite uniform particles of polyhedral shape. Particle size and size distribution was estimated from several SEM micrographs using ImageJ software. It was determined that particles vary from 0.3 to 1.6 µm in size and 86% of all particles are in the range from 0.6 to 1.2 µm. It should be noticed that these particles tend to aggregate into larger agglomerates, which are clearly seen in Figure 3b.

**Figure 4:**
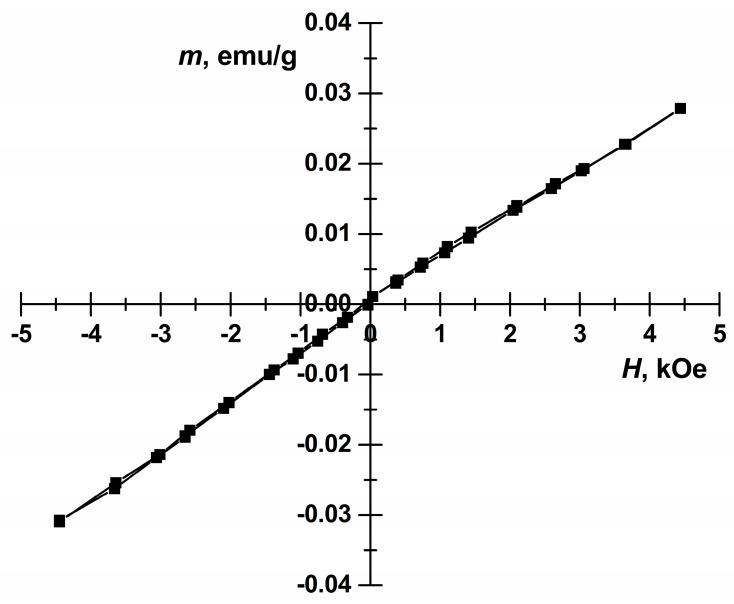
Magnetic hysteresis of BFO powders annealed at 650 °C.

Room temperature dependence of magnetization of BFO powders annealed at 650 °C on applied magnetic field strength is illustrated in Figure 4.

**Figure 5:**
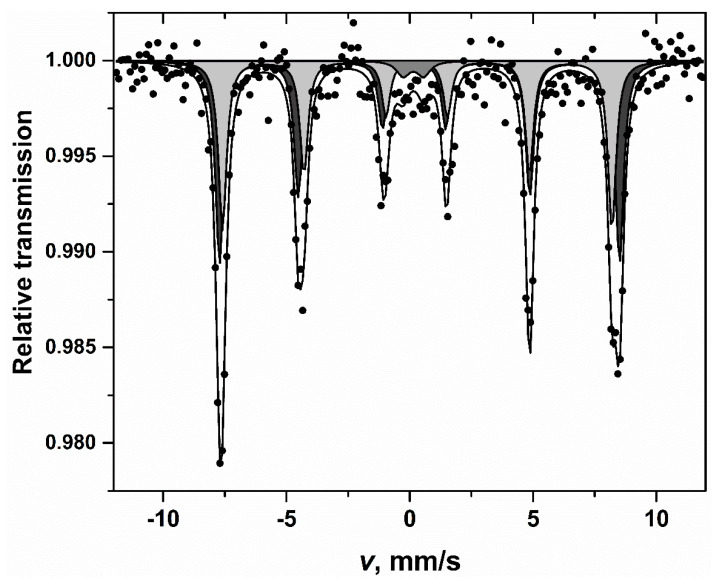
Mössbauer spectrum of BFO powders recorded at room temperature.

Linear dependence of magnetization of the sample is typical of antiferromagnetic materials and characteristic of bulk BFO [34]. In this case magnetization arising due to non-compensated magnetic moments at surface of particles is insignificant as particle sizes are much larger than the period length of spin modulation of 62 nm [35]. The Mössbauer spectrum of BFO powder annealed at 650 °C is presented in Figure 5.

Two sextets corresponding to antiferromagnetic BFO and low intensity (≈ 5% of area) paramagnetic/superparamagnetic doublet were fitted to Mössbauer spectra (Figure 5, Table 1). The doublet may correspond to Fe in paramagnetic Bi_2_Fe_4_O_9_ [36]. The isomer shift of 0.25 ± 0.09 mm/s which is lower than those of sextets can be due to the influence of Fe tetrahedral coordination. The asymmetry and line broadening characteristic of BFO Mössbauer spectra was explained by dependence of hyperfine field *B* and quadrupole shift 2ε on the angle *θ* between *c* crystalline axis, which is also principal axis of the electric field gradient (EFG) and the direction of Fe spin [34,37]. Because of spiral magnetic structure Fe spin direction changes from parallel to perpendicular to *c*-axis of BFO. Two sextets are sufficient to achieve good fitting quality of Mössbauer spectra taking into account the variation of *B* and 2ε.

**Figure 6:**
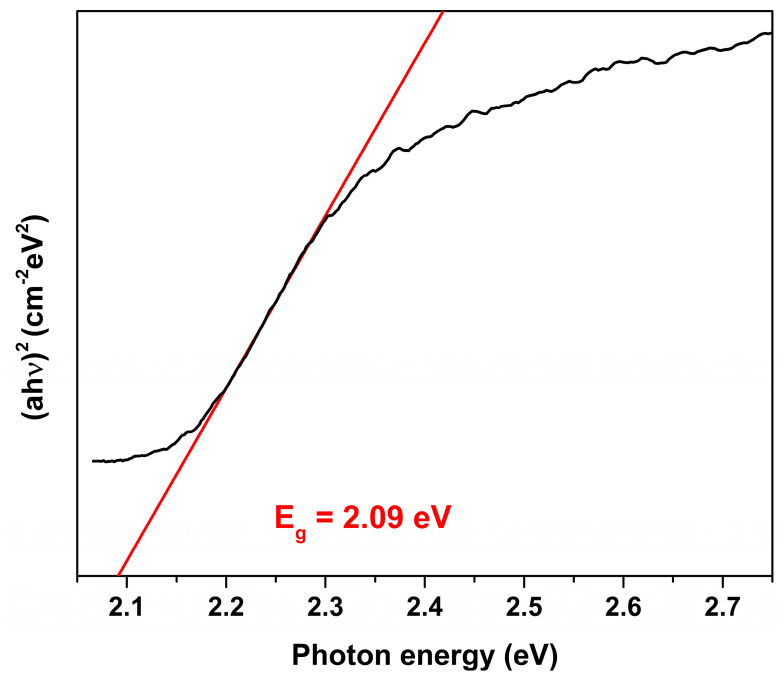
Plot of (ahν)^2^ versus photon energy for BFO powder annealed at 650 °C.

In order to estimate an optical band gap of the synthesized BFO powders, UV-Vis diffuse reflection spectrum was recorded and recalculated employing the Kubelka-Munk function [38]. Figure 6 illustrates the calculation of *E_g_* using Tauc equation, (*ahv*)*^n^*= *C*(*hv−E_g_*), where *a* is absorption coefficient, *h* is Planck constant, *ν* is light frequency, *E_g_* is band gap energy and *C* is a constant.

The value of *n* was chosen as 2, which corresponds to a direct band gap. As shown, the *E_g_* value is estimated by extrapolating the linear part of (ahν)^2^ against the hv plot to the point a = 0. The calculated *E_g_* value was 2.09 eV, which suggests that synthesized BFO powders effectively absorb visible light in the range of 380–593. Thus, the synthesized BFO powders exhibited a strong potential for the photocatalytic application for the degradation of organic pollutants using the visible light. This value is in a good agreement with *E_g_* previously reported for BFO powders [39,40]; however, it is lower in comparison to that of BFO thin films [41]. Such difference could be explained by size effect and caused by the small thickness of the films.

## 4. Conclusions

Highly crystalline BFO powders were synthesized employing a novel, simple and cost-effective synthetic approach. Despite the fact that complete thermal decomposition of BFO precursor gel occurred at 450 °C, it was determined that optimal annealing temperature for the preparation of phase pure BFO is 650 °C. At lower or higher temperatures formation of undesired secondary phases was observed. The diffraction peaks of BFO XRD pattern were very sharp and well-resolved indicating high degree of crystallinity of the powders. The synthesized powders under external magnetic field exhibited linear dependence of magnetization, which is typical of antiferromagnetic materials and characteristic of bulk BFO. Optical band gap was estimated on the basis of diffuse reflectance spectra of the BFO powders and calculated *E_g_* value was 2.09 eV.

## Figures and Tables

**Table 1:** Mössbauer spectra parameters. *S*, *Γ*, *δ*, 2ε (Δ), *B* are relative intensity, line width, isomer shift relative to α-Fe at room temperature, quadrupole shift (splitting) and hyperfine field, respectively.

*S*, %	*Γ,* mm/s	*δ,* mm/s	2ε(Δ)*,* mm/s	*B*, T
53 ± 6	0.4 ± 0.03	0.39 ± 0.01	0.22 ± 0.02	50.28 ± 0.08
42 ± 6	0.4 ± 0.04	0.40 ± 0.01	0.02 ± 0.02	48.97 ± 0.10
5 ± 2	0.6 ± 0.2	0.25 ± 0.09	0.83 ± 0.16	-
